# Gestational low-protein diet impairs mitochondrial function and skeletal muscle development by inducing immune responses in male offspring

**DOI:** 10.1016/j.redox.2025.103890

**Published:** 2025-10-10

**Authors:** Atilla Emre Altinpinar, Moussira Alameddine, Ufuk Ersoy, Ioannis Kanakis, Vanja Pekovic-Vaughan, Susan E. Ozanne, Katarzyna Goljanek-Whysall, Aphrodite Vasilaki

**Affiliations:** a Department of Musculoskeletal and Ageing Science, Institute of Life Course & Medical Sciences (ILCaMS), The MRC - Versus Arthritis Centre for Integrated Research into Musculoskeletal Ageing (CIMA), University of Liverpool, Liverpool, UK; b Chester Medical School, Faculty of Medicine and Life Sciences, University of Chester, Chester, UK; c MRC Metabolic Diseases Unit and Institute of Metabolic Science – Metabolic Research Laboratories, Institute of Metabolic Science, Addenbrooke's Treatment Centre, Addenbrooke's Hospital, University of Cambridge Metabolic Research Laboratories, Cambridge, UK; d Department of Physiology, School of Medicine and REMEDI, CMNHS, University of Galway, Galway, Ireland

**Keywords:** Maternal nutrition, Protein restriction, Skeletal muscle, Immune response, Inflammation, Oxidative stress

## Abstract

Maternal nutrition is essential for proper fetal and postnatal organ maturation and is linked to the future risk of developing metabolic syndrome, cardiovascular disease, and muscle loss. There is still limited understanding how a low-protein intake during gestation influences skeletal muscle development, inflammation, and the related pathways. This study aimed to investigate the impact of gestational low-protein diet in mice on skeletal muscle development and inflammatory responses in male offspring.

Pups born from mothers fed a low-protein diet (LPD) were lactated by normal protein diet (NPD)-fed mothers and maintained on NPD post-weaning (LNN group). Offspring born from mothers fed an NPD and maintained on an NPD during lactation and beyond were used as controls (NNN group).

In 21-day-old offspring from protein-restricted mothers, RNA-Seq analysis showed upregulation of immune response–related genes, enriching adaptive immunity pathways. Additionally, LNN group exhibited elevated markers of inflammation, along with disruptions in antioxidant defence balance and macrophages infiltration in gastrocnemius muscle at 3 months of age. Energy metabolism was impaired, as indicated by changes in related proteins and enzymes involved in mitochondrial function.

We conclude that gestational LPD adversely affects skeletal muscle development in male offspring.

## Highlights

• Gestational protein restriction induces an offspring immune response.
• Gestational protein restriction leads to decreased fibre number in offspring muscle.
• Gestational protein restriction disrupts muscle energy metabolism.
• Gestational protein restriction increases muscle oxidative stress.

## 1 Introduction

Maternal nutrition is a fundamental environmental factor in regulating fetal and postnatal offspring organ development, and plays a crucial role in influencing the risk of developing metabolic and cardiovascular diseases, as well as sarcopenia later in life [[1], [2], [3]]. Maternal nutrient restriction during pregnancy can lead to the birth of small-for-gestational-age (SGA) or intrauterine growth-restricted (IUGR) offspring, which have been linked to an increased risk of poor cardio-metabolic health in adulthood [2,[4], [5], [6], [7], [8]]. Among various forms of nutrient deficiencies, maternal protein deficiency (MPD) remains a significant concern in populations worldwide [9,10].

Skeletal muscle development is highly sensitive to nutritional deficiencies during the gestational period [11]. Skeletal muscle fibres form during two critical phases—primary and secondary myogenesis—both occur during prenatal development [12,13]. The total number and composition of muscle fibres formed during fetal life are key determinants of future muscle integrity and function [14]. Studies have demonstrated that an isocaloric maternal or lifelong low-protein diet alter offspring skeletal muscle development by disrupting growth patterns and mitochondrial homeostasis [15], decreases fibre number and muscle strength [16], impairs insulin signalling, and contributes to an ageing-like phenotype in skeletal muscle [17,18]. Therefore, proper maternal nutrition plays a pivotal role in establishing optimal structural and functional maturation of skeletal muscles [17,[19], [20], [21]]. However, the exact mechanisms underlying the effects of gestational protein restriction on skeletal muscle development and growth in male offspring still remain understudied.

Maternal low-protein intake during pregnancy has been associated with changes in gene expression in the skeletal muscle of new-born offspring [22]. Gestational protein restriction promotes accelerated ageing, increased DNA damage, oxidative stress, enhanced antioxidant responses, and inflammation, potentially mediated by Nuclear factor kappa B (NF-κB) signalling in adult male offspring [17]. Inflammatory responses triggered by nutritional deficiencies [21,23] are recognised as one of the key mechanisms of skeletal muscle loss and mitochondrial dysfunction and elevated reactive oxygen species (ROS) levels [24]. Inflammation in skeletal muscle triggers macrophage infiltration, which plays a pivotal role in initiating and supporting muscle regeneration [25]. Moreover, inflammation has been shown to interfere with mitochondrial biogenesis and dynamics, primarily by downregulating regulators such as Peroxisome proliferator-activated receptor gamma coactivator 1-alpha (PGC-1α), leading to mitochondrial dysfunction and impaired ATP production [26].

Here, we examined the impact of a gestational low-protein diet on skeletal muscle development, redox homeostasis, and inflammatory signalling in weaned and young/adult male offspring. Our findings revealed that prenatal protein restriction leads to an upregulation of adaptive immune-related genes at a postnatal stage (21-days-old), a reduction in muscle fibre number, and alterations in fibre type composition by 3 months of age in male offspring, suggesting that a gestational low-protein diet potentially predisposes them to impaired growth and, altered metabolism.

## 2 Materials and methods

### 2.1 Animals

For this study, B6.Cg-Tg(Thy1-YFP)16Jrs/J mice (Jackson Laboratory; stock number 003709) were utilised. The mice were housed in individually ventilated cages under controlled conditions with a fixed light/dark cycle (21 ± 2 °C, 12-h light/12-h dark cycle). Ethical approval for the study was granted by the University of Liverpool Animal Welfare Ethical Review Committee (AWERB), and all experimental protocols involving the handling and use of laboratory animals were conducted in accordance with the UK Animals (Scientific Procedures) Act 1986 (PPL code: P4A0493B2; approval date: 26 March 2019).

### 2.2 Generation of experimental groups

**Fig. 1:**
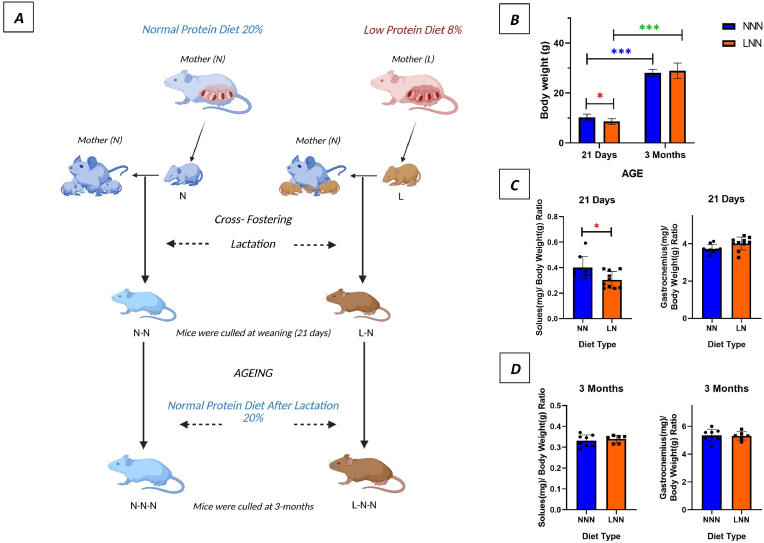
**Experimental design and changes in phenotypic characteristics, muscle and body weights of male offspring as a result of gestational low protein diet.** **(A)**: (N) a 20 % protein diet and (L) 8 % protein diet fed mothers. **Group N**: Control group litters obtained from dams fed on 20 % protein diet chow. **Group L**: Experiment group litters obtained from dams fed on an 8 % protein diet chow. **Group NN**: Mice obtained from dams fed a normal protein diet and maintained on a normal diet after birth. They were culled and analysed at weaning (21 days). **Group LN**: Mice were born from dams fed a low protein diet, but lactated by a mother maintained on a normal diet. They were culled and analysed at weaning (21 days). **Group NNN:** Control group mice were born from dams fed on a normal protein diet, were lactated by a mother maintained on a normal diet, and fed on a normal diet until 3 months of age. **Group LNN**: Experimental group mice were born from dams fed on a low protein diet, were lactated by a mother maintained on a normal diet, and fed on a normal diet until 3 months of age. **(B)** Comparison of total body weight (g) at different ages and diet types for 21 days and 3 months. Comparison of soleus and gastrocnemius weights to body weight ratio (g) at different ages and diet types for 21 days and 3 months **(C**–**D)**. Data are presented as mean ± SD. Student’s t-test and Mann-Whitney U were used to determine statistical significance. ∗ Indicates p < 0.05, ∗∗ indicates p < 0.01, ∗∗∗ indicates p < 0.001 (n = 9–10, 21 days), (n = 6–8, 3 months) (Created in BioRender. ALTINPINAR, A. (2025) https://BioRender.com/rkjh2w0).

Two weeks prior to mating, nulliparous 8-weeks old female mice were fed either a normal-protein diet (N, 20 % crude protein; code 824226, Special Diet Services, UK) or a low-protein diet (L, 8 % crude protein; code 824248, Special Diet Services, UK) ad-libitum. Male mice of the same age, which had been fed on a normal-protein diet, were used for mating. The male pups born from both groups were used for this study. New-born offspring were cross-fostered to dams fed normal-protein diet within 24 h of birth, creating Normal-Normal (NN) and Low-Normal (LN) groups. NN and LN mice were kept on a normal-protein diet after weaning (day 21), generating Normal-Normal-Normal (NNN) and Low-Normal-Normal (LNN) groups of mice respectively. Mice had ad-libitum access to food and water. Mice were culled at weaning (21 days; n = 9–10) and at 3 months of age (n = 6–8) (Fig. 1 A). Prior to dissection, body weights were measured, and the soleus (SOL) and gastrocnemius (GAS) muscles were dissected and weighed.

### 2.3 Histological analysis

Soleus muscles were dissected and positioned vertically on cork discs. Muscles were embedded in Optimal Cutting Temperature (OCT – Thermo Fisher Scientific, Waltham, MA, USA) compound and immersed in isopentane (Sigma, Poole, UK) cooled by liquid nitrogen. Transverse sections (8–10 μm thick) were sectioned using a Leica 1950 (Leica Biosystems, Newcastle upon Tyne, UK) for histological analysis.

### 2.4 Immunohistochemistry

Sections were air-dried for 30 min and then washed with phosphate buffered saline (PBS) before being fixed with 100 % ice-cold acetone for (Myosin Heavy Chain (MYH), Wheat Germ Agglutinin (WGA), and CD68 staining). Cryosections were blocked for an hour at room temperature with MYH and WGA, using a Mouse on Mouse (M.O.M) blocking kit (Vector Laboratories Ltd., Peterborough, UK) in 5 % Goat Serum in Phosphate Buffered Saline (PBS). For CD68, a blocking solution containing 15 % goat serum, 0.25 % Triton X-100, and 2 % bovine serum albumin (BSA) (Sigma-Aldrich Ltd., Gillingham, Dorset, UK) in PBS was used.

Cryosections were incubated with primary antibodies in 5 % Goat Serum in PBS overnight at 4 °C, followed by incubation with secondary fluorescent antibodies (Invitrogen) for 1 h at room temperature. VECTASHIELD Antifade Mounting Medium (Vector Laboratories Ltd., Peterborough, UK) was utilised to mount sections, and images were captured with a Zeiss LSM 800 confocal microscope (Zeiss, Oberkochen, Germany). Images were analysed using ImageJ (U.S. National Institutes of Health, USA) and MyoVision software (University of Kentucky, USA). Cellpose 2.0 used for segmentation of fibre specific Cross-Sectional Area (CSA) analysis.

### 2.5 Haematoxylin & eosin staining

Samples were air dried for 30 min, hydrated with PBS, fixed with 4 % paraformaldehyde (PFA) for 2 min, and washed with PBS for 5 min twice. They were then transferred to a staining jar with Harris haematoxylin (Leica) solution for 45 s, washed with running tap water for 30 s, transferred in a staining jar with Eosin Y (Leica) solution for 15 s, and washed with running tap water for 30 s. The slides were next transferred into staining jars with 70 % ethanol, 90 % ethanol, 100 % ethanol for dehydration 30 s respectively, and cleared with xylene for 30 s, and then air-dried slides mounted with Histomount (National Diagnostics, Charlotte, NC, US). Nikon Ci (Nikon, Kingston, UK) used to capture images.

### 2.6 RNA extraction, library preparation and sequencing

Frozen GAS muscles dissected from 21-day-old mice were powdered using a pestle and mortar in liquid nitrogen. Total RNA was extracted and purified from frozen powdered GAS muscle using RNeasy mini kits with on-column DNase treatment (Qiagen, Manchester, UK), in accordance with the manufacturer's instructions. The preparation of a dual-indexed, strand-specific RNA-Seq library from the submitted total RNA was carried out using the NEBNext polyA selection and Ultra II Directional RNA library preparation kits (New England Biolabs, UK), as previously described [15]. The integrity of total RNA was verified using an Agilent 2100 Bioanalyzer (Agilent Technologies, Santa Clara, CA, USA), achieving an RNA Integrity Number (RIN) greater than 7. RNA-Seq was performed on an Illumina NovaSeq using S1 chemistry (paired-end, 2 × 150 bp sequencing), which generated approximately 650 million clusters per lane.

### 2.7 Data processing and bioinformatic analyses

The Centre for Genomic Research at the University of Liverpool processed the raw Fastq files by trimming Illumina adapter sequences using Cutadapt version 1.2.1. [27]. Reads that match the adapter sequence for at least 3 base pairs were trimmed. Sickle version 1.200, with a minimum window quality score of 20, was utilised to trim the reads, and those shorter than 15 bp after trimming were removed. The trimmed reads were subsequently pseudo-aligned to the Mus musculus transcriptomes from Ensembl release v96 using Kallisto quant version 0.46.1 to obtain transcript length and abundance estimates [28]. The yield from these tools was transformed into count data using tximport (TXI) version 1.26.1 in R version 4.2.2. [29]. DESeq2 version 1.38.3. 3.4.1 in R used for differential expression analysis. In comparison of gene expressions, cutoffs have been used as |Log2 Fold Change| (|Log2FC|)>0.3 and adjusted p-values<0.1. Next, potential biological pathways and enrichment analysis were performed by using ShinyGO version 0.81 (FDR<0.05) [30]. All up-regulated and down-regulated gene names were entered into ShinyGO separately and mouse selected as species. The network image is created by using the STRING 12.0 version.

### 2.8 Gene expression analyses

GAS muscles were powdered using a mortar and pestle immersed in liquid nitrogen. Total RNA was subsequently extracted employing the TRIzol method (Thermo Fisher Scientific, Waltham, MA, USA), followed by purification using RNeasy mini kits. RNA concentration and purity were assessed using the NanoDrop 2000 (Thermo Fisher Scientific, Waltham, MA, USA). A quantity of 1000 ng of mRNA was utilised for cDNA synthesis employing a High-Capacity cDNA Reverse Transcription kit. Subsequently, RT-qPCR was conducted using 10 ng of cDNA and SYBR Hi-ROX Kit with a reaction volume of 10 μL. The acquired data were analysed using LightCycler 96 version 1.1 software (Roche Diagnostics, 2011).

Expression levels were normalised to 18S rRNA at both 21-days-old and 3 months of age, as the expression of this reference gene remained consistent across groups at each respective age (supplementary figure-3). The relative fold change in gene expression was calculated using the delta-delta Ct method (2–ΔΔCt) [31]. All primers were validated before conducting gene expression analysis, and their sequences are provided in supplementary table-1.

### 2.9 Western blotting

Total protein extracted from powdered GAS muscles. RIPA Lysis buffer (Sigma, Poole, UK) that contains Protease and Phosphatase Inhibitor Mini tablets (Thermo Fisher Scientific, Waltham, MA, USA), Ethylenediaminetetraacetic acid (EDTA)-free. Samples were homogenised and then sonicated twice for 30 s each. Samples were centrifuged, and the supernatant was collected. To determine the concentration of samples, BCA Protein assay was used. Protein extracts of 30–40 μg were subjected to electrophoresis and separated on NuPAGE™ 4–12 % Bis-Tris Mini Protein Gels with a thickness ranging from 1.0 to 1.5 mm (Invitrogen, Renfrewshire, UK). Following electrophoresis, the proteins were transferred onto a polyvinylidene fluoride (PVDF) membrane (Sigma, Poole, UK) and 5 % bovine serum albumin (BSA) (Sigma, Poole, UK) in tris-buffered saline (TBS) was used to block the membrane for a 1 h at room temperature. Membranes were incubated with primary antibody at 4 °C overnight and then incubated with secondary antibodies at room temperature for 1 h (Licor, Bad Homburg, Germany). The blots were imaged using the Licor Odyssey CLx system, and band densities were subsequently analysed utilising Image Studio™(Licor) software. The obtained results were normalised to the loading control (GAPDH or Ponceau (Thermo Fisher Scientific, Waltham, MA, USA)).

OxiSelect™ Protein Carbonyl Immunoblot Kit (Cell Biolabs, San Diego, California, United States) was used to detect Protein carbonyls, which directly derivatizes proteins on the membrane after separation by SDS-PAGE and transfer. PVDF membranes were utilised and derivatised according to the manufacturer's protocol. 5 % BSA (Sigma, Poole, UK) was used to block the membrane in TBS-Tween 20 for 1 h at room temperature and incubated with anti-Dinitrophenyl (DNP) (Cell Biolabs 1:1000) in 5 % BSA TBS-T overnight at 4 °C. The membrane was incubated with Licor secondary antibodies (Licor, Bad Homburg, Germany), and the blots were imaged with the Licor Odyssey CLx. Image Studio™(Licor) software was used to analyse band densities. The membrane was subsequently stripped, washed, blocked, and incubated with a primary antibody targeting the house-keeping protein GAPDH. The results were then normalised to GAPDH signal intensity.

### 2.10 Antibodies

The primary antibodies used for Western blotting and Immunohistochemistry are listed below, along with their corresponding dilutions (Table 1).

**Table 1:** **List of primary antibodies used in this study.** This table summarises all primary antibodies used in the study, including, supplier information, catalogue numbers, dilutions, and applications. All dilutions refer to those optimized and used in the described experiments.

Antibody	Catalogue no.	Dilution	Application	Source
GAPDH	ab8245	1:3000	Western Blot	Abcam (Cambridge, UK)
Anti-PGC1 alpha	ab191838	1:750	Western Blot	
Citrate synthase	ab129095	1:1000	Western Blot	
Anti-HO-1	ab13243	1:500	Western Blot	
Anti-CD68	ab125212	1:500	Western Blot	
		1:200	Immunohistochemistry	
Anti-p62	ab91526	1:1000	Western Blot	
Anti-AMPKα	#2532	1:500	Western Blot	Cell Signalling (Cell Signalling Technology, Danvers, MA, USA)
Anti- Phospho-AMPKαThr172	#2535	1:500	Western Blot	
Anti-NF-κB p65	#8242	1:750	Western Blot	
Anti-LC3B	43566	1:400	Western Blot	
Anti TNF-α	#11948	1:1000	Western Blot	
Anti-Caspase 3	#9662	1:750	Western Blot	
Anti-SOD2	ADI-SOD-111	1:2000	Western Blot	Enzo Life Sciences (Farmingdale, New York, USA)
Anti-Dystrophin	(MANDRA1(7A10)	1:20	Immunohistochemistry	DSHB (Iowa, USA)
Anti-MYH I	BA-D5	1:200	Immunohistochemistry	
Anti-MYH IIa	SC-71	1:250	Immunohistochemistry	
Wheat Germ Agglutinin (WGA)	CF®405S Conjugate	1:500	Immunohistochemistry	Generon (Farmingdale, New York, USA)

### 2.11 Statistical analyses

For statistical analyses, the Shapiro-Wilk normality test, Student's *t*-test, and Mann-Whitney *U* test were performed using GraphPad Prism software (version 8.0.2). All data is presented mean ± standard deviation (SD). Statistical significance is considered p < 0.05. n demonstrates the number of mice(litters) used per group.

## 3 Results

### 3.1 Lower body and skeletal muscle weights in 21-day-old mice but a compensatory growth in 3-month-old male offspring mice on a gestational low protein diet

This study examined the effects of a gestational low-protein diet on male offspring born to mothers fed either an 8 % isocaloric or 20 % standard protein diet (Fig. 1- A). At weaning, 21 day-old low-protein (LN) mice had a significantly lower body weight and soleus-to-body weight ratio (p < 0.05), while the GAS muscle weight-to-body weight ratio remained similar across groups (Fig. 1- B, C, D). 3-month-old LNN mice displayed compensatory growth, with a slightly higher body weight and a slightly lower soleus-to-body weight ratio compared to NNN control mice, though these differences were not statistically significant (p > 0.05) (Fig. 1- B, C, D).

### 3.2 Gestational protein restriction alters skeletal muscle composition, size and structure in male offspring mice

**Fig. 2:**
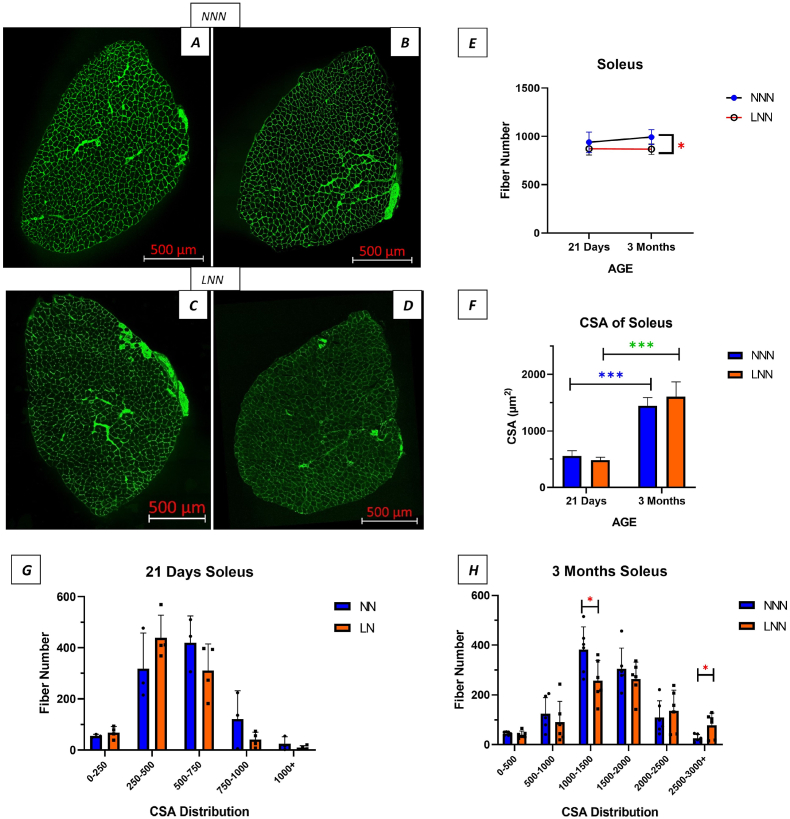
**Gestational protein restriction alters muscle fibre number, and CSA in soleus muscles in 21 days and 3 months old male offspring.** Representative confocal images of soleus muscles stained with wheat germ agglutinin (WGA; green) (Rhodamine) (3 Months NNN; **A-B,** LNN **C-D**). **Graph** (**E):** Comparison of fibre number at different ages and diet types at 21 days and 3 months. **Graph** (**F):** Comparison of CSA at different ages and diet types at 21 days and 3 months. **Graph** (**G-H):** Comparison of fibre CSA distribution at 21 days and 3 months respectively. ∗ Indicates p < 0.05, ∗∗∗ indicates p < 0.001. (n = 3–4, 21 days), (n = 6, 3 months). Magnification 20x, Scale bars = 500 μm.

To further investigate the effects of lower body weight-to-soleus weight ratio in 21-day-old mice and subsequent catch-up growth at 3 months, histological analyses were performed on WGA-stained soleus muscle sections. Fibre number and cross-sectional area (CSA) were assessed. LN mice at 21 days exhibited a trend towards a lower fibre number and CSA (p > 0.05) (Fig. 2- E, F). At 3 months, LNN mice showed a significantly lower fibre number (p < 0.05) with a slightly higher CSA, though the latter was not statistically significant (p > 0.05) (Fig. 2- E, F). In the protein-restricted group, fibre CSA distribution shifted from smaller fibres at 21 days to more large fibres by 3 months, compared with controls. (Fig. 2- G, H).

**Fig. 3:**
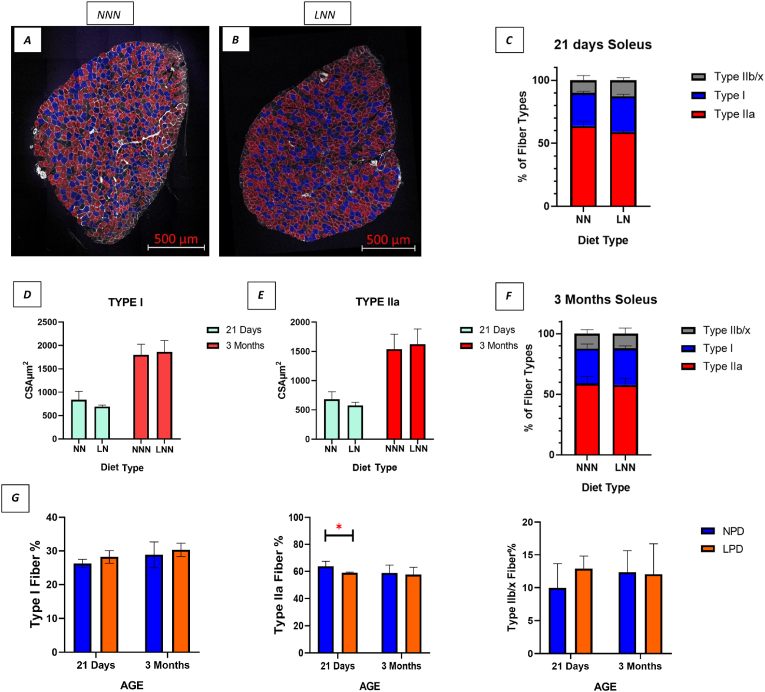
**Changes in fibre type and, fibre type specific CSA in soleus muscles in 21 days and 3 months old male offspring following gestational protein diet.** Representative confocal images of soleus muscles stained for antibodies against different muscle fibre types (red; Type IIa), (Blue; Type I), (Grey; Type IIb/x) (3 Months NNN; **A,** LNN; **B**). **Graph (C–F):** Composition of soleus muscle fibre types in 21 days and 3 months offspring. **Graph** (**D-E):** Comparison of muscle fibre specific CSA in two age groups. **Graph** (**G):** Comparison of muscle fibre types; Type I, Type IIa and Type IIb/x respectively. ∗ Indicates p < 0.05. (n = 3–4, 21 days), (n = 6, 3 months). Magnification 20x, Scale bars = 500 μm.

Next, muscle fibre type distribution was evaluated in soleus muscle sections stained with antibodies for different muscle fibre types. Type I fibre percentages were slightly higher in LPD mice at both time points, though the difference was not statistically significant (Fig. 3- G). Notably, the proportion of type IIa fibres was significantly lower in 21-day-old LN mice (p < 0.05), but this difference was resolved by 3 months (Fig. 3- G). Type IIb/x fibre proportions were initially higher at 21 days in LN mice but equalized by 3 months (p > 0.05) (Fig. 3- G). Graphs showing the fibre type compositions of soleus muscle at 21 days and 3 months of age are also presented as a whole. It was observed that both groups consisted of 57–60 % type IIa, 25–31 % type I, 9–12 % type IIb/x fibres. (Fig. 3- C, F). Additionally, the CSAs of type I and type IIa fibres were measured at both ages. While type I and type IIa fibres had a tendency for a smaller CSA at 21 days in LNN mice, CSA were similar between the NNN and LNN groups at 3 months (p > 0.05) (Fig. 3- D, E).

### 3.3 Upregulation of immune-related genes in the skeletal muscle of 21 day-old male offspring on a gestational protein restriction

**Fig. 4:**
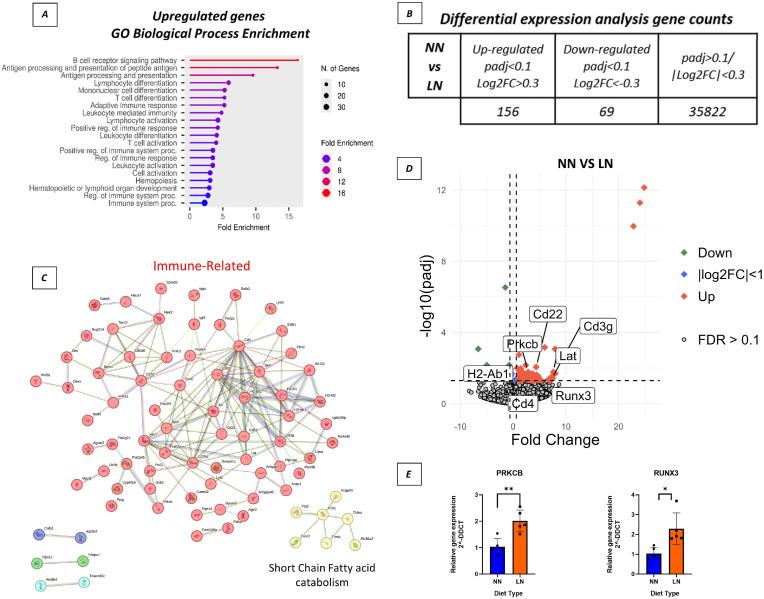
**Low-protein diet during gestation upregulates adaptive immune response related genes in the gastrocnemius muscles of 21-day-old male offspring.** **(A):** Gene ontology (GO) biological process enrichment analysis of upregulated genes **(NN vs LN (n = 6)). (B)** Differential expression analysis gene counts **(C):** Gene interaction networks show immune related genes (red), and short chain fatty acid catabolism related genes (yellow) constructed using STRING. **(D):** The volcano plot showing differentially expressed genes (Upregulated = orange, downregulated = green) (p adjusted <0.1, log2FC > 1) **(E):** qPCR analysis confirming the expression levels of selected upregulated genes (n = 5). Data are presented as mean ± SD. ∗ Indicates p < 0.05, ∗∗p < 0.01.

To examine the effects of a gestational low protein diet on gene regulation and to identify affected pathways through global profiling, RNA-Seq was performed at the end of the lactation period from gastrocnemius muscles in NN and LN 21-day-old male mice. Firstly, differential expression analysis indicated that **156 genes were found to be upregulated**, while **69 genes were downregulated** (Fig. 4 -B). When the upregulated and downregulated genes were separately uploaded for Gene Ontology Biological Process (GO-BP) enrichment analysis, mostly immune response pathways were significantly enriched among the upregulated genes, such as the GO:0002250 **adaptive immune response** ((enrichment False Discovery Rate) FDR< 0.00047), GO:0042110 **T cell activation** (FDR< 0.004), and GO:0050853 **B cell receptor signalling pathway** (Fig. 4 -A). Key upregulated genes included **Cd3g, Cd79a, Kit, Cd4, Lat, Cd3d, Ly6d, Lat2, Igf2, H2-Eb2, H2-Ab1, Foxp1, Vav1, Pou2f2, Satb1, Il18r1, Pck1, Ezh2, Prkcb, and Runx3** which play essential roles in immune-related pathways. These enrichments suggest a robust activation of adaptive immunity in response to gestational nutritional restriction. Analysis of the downregulated genes revealed no significant enrichment in KEGG pathway database.

In light of these findings, STRING was used to analyse the interactions and clustering patterns between the upregulated genes. The analysis revealed two major clusters; 68 of the 156 upregulated genes were immune-related (coloured-red), 7 of the 156 were related to short-chain fatty acid catabolism (coloured-yellow) and their connections were visualised (Fig. 4 -C). Furthermore, differential gene expression analysis in RNA-Seq data was visualised using the volcano plot, and some of the immune-related genes were highlighted (Fig. 4 -D). PRKCB (Protein Kinase C Beta) and RUNX3 were randomly selected among 68 immune-related genes for validation as shown on the plot. Finally, rt-qPCR was performed to confirm RNA-Seq data and showed that these genes, PRKCB (P < 0.01) and RUNX3 (p < 0.05), were significantly upregulated (Fig. 4 -E).

### 3.4 Increased macrophage infiltration, oxidative stress markers and inflammation in 3-month-old male offspring on gestational low protein diet

The changes in adaptive immune related genes at 21 days in male offspring strongly indicate an active immune response due to gestational low protein diet. Therefore, we next analysed the potential downstream impacts of activated immune response in skeletal muscles such as macrophages infiltration.

**Fig. 5:**
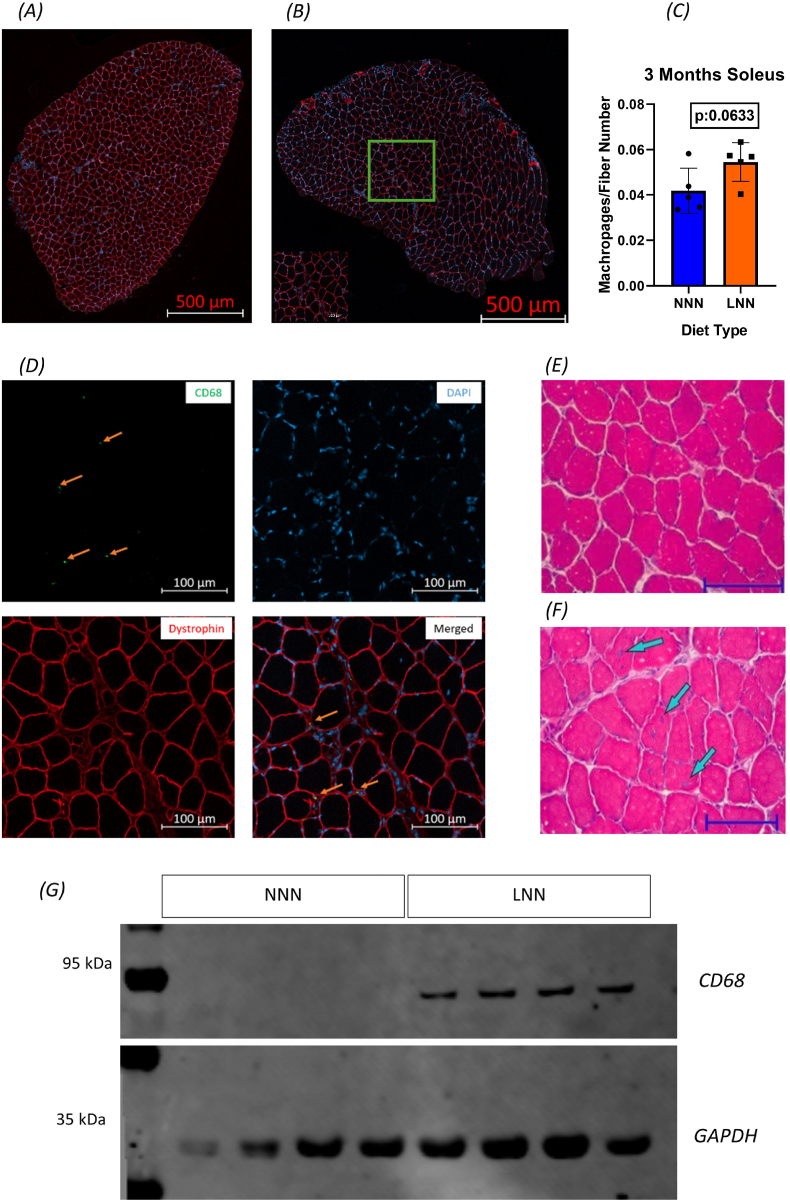
**Gestational protein restriction increases macrophages infiltration and the number of centralised nuclei in 3-month-old offspring male mice** Representative confocal images of soleus muscles stained with dystrophin (red), CD68 (green), DAPI (blue), (3 Months NNN; **A,** LNN **B, n = 5**). Statistical analysis of macrophage number to muscle fibre number ratio at 3 months of age (**C**). Representative individual micrograph channels that show dystrophin (red-muscle fibres), CD68 (green- macrophages), DAPI (blue-nuclei), and merged in LNN muscles (**D**). Representative H&E images of 3 months soleus (**E**-NNN, **F**-LNN blue arrows shows the centralised nuclei) (n = 5, 3 months)). Magnification 20x, Scale bars = 500 μm (**A-B**), 100 μm (**D-E-F**). Student’s t-test used as a statistical test. (**G**) Western blot results for CD68 and GAPDH (Loading Control; Gastrocnemius muscle).

In LNN group of 3-month old male mice, the ratio of macrophages to muscle fibres was elevated compared to the NNN group, which fell short of significance (p < 0.1) (Fig. 5- C), prompting further investigation into proteins associated with oxidative stress and inflammation. Western blotting analysis of the GAS muscles revealed detectible CD68 protein levels in the LNN group (Fig. 5 - G). No statistical analysis was performed, due to no detectable protein bands were observed in the control group. Moreover, centralised nuclei, which mark muscle fibres undergoing cycles of damage/regeneration, were also observed to be elevated in the LNN group (Fig. 5- D, E, F). In this experiment (n = 5), no central nuclei were observed in the 3 NNN mice, while they were observed in all mice in the LNN group, and when averaged, they were higher; however, no statistical test was used.

**Fig. 6:**
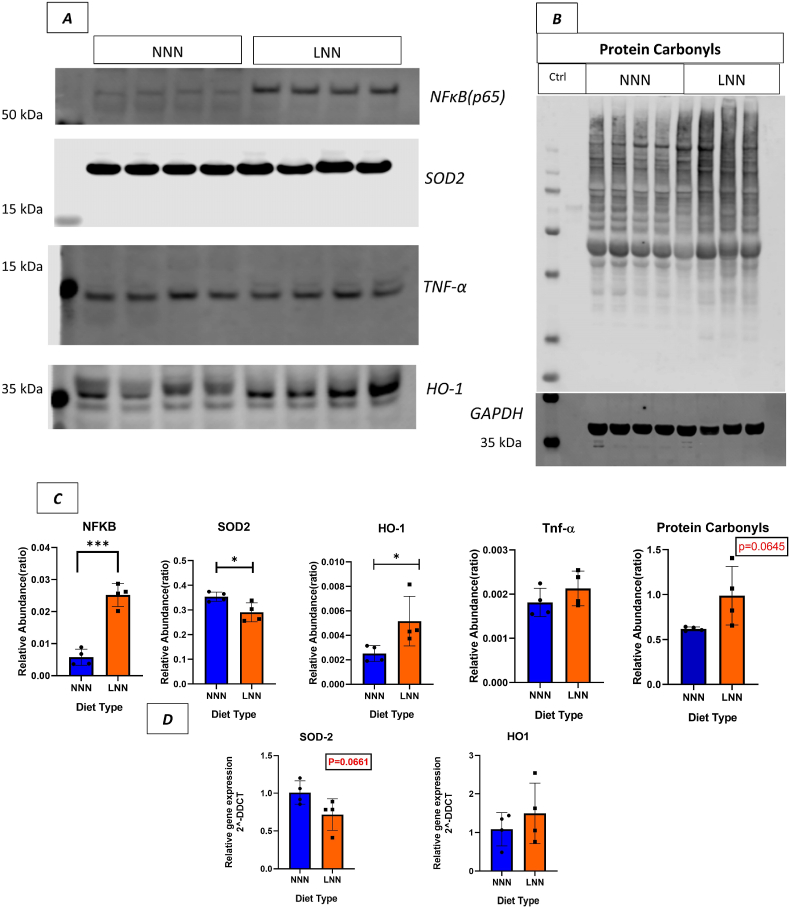
**Gestational protein restriction affects oxidative stress and inflammation markers in 3-month-old offspring male mice.** **(A)** Western blot results for Nuclear factor-κB (NF-κB), Superoxide Dismutase (SOD2), Tumour Necrosis Factor alpha (TNF-α), heme oxygenase-1 (HO-1) **(GAS muscle) n = 4**. **(B)** Protein Carbonyl levels detected by western blotting and GAPDH was used as loading control. **(C)** Quantification and statistical analysis of protein relative abundance ratio in graphs (NF-κB, SOD2, Protein Carbonyl). Ponceau **(for SOD2, TNF-** α, **HO-1)** (supplementary figure 1-A, Fig. 2-B,C) or GAPDH **(for NF**κ**B)** (Supplementary Fig. 1–B) staining were used as loading controls. **(D)** Statistical analysis of gene expression levels in SOD2 and HO-1 genes. ∗ indicates p < 0.05, ∗∗∗ indicates p < 0.001. Student’s t-test used as a statistical test.

Next, we examined oxidative stress and inflammatory markers using western blotting. Notably, in the LNN group, a significant reduction in mitochondrial Superoxide Dismutase 2 (SOD2) relative protein abundance was detected (p < 0.05) as well as a near-significance in the reduction of its gene expression levels (Fig. 6- A, C, D). In contrast, Heme Oxygenase (HO-1) relative protein abundance was increased in LNN group (p < 0.05), with a trend towards increased gene expression level, suggesting an altered redox balance (Fig. 6- A, C, D). Moreover, even though there was a slightly increase but not significant (p > 0.05) change in the relative protein abundance level of Tumour Necrosis alpha (TNF- α), a significant increase in Nuclear Factor-κB (NF-κB) protein abundance was observed (p < 0.001) in the LNN group, indicating increased immune and inflammatory responses (Fig. 6- A, C). Concurrently, protein carbonyl levels (recognised biomarkers of oxidative damage) showed an upward trend in the LNN group (p < 0.1). These findings collectively suggest an altered homeostasis of oxidative and inflammatory markers in the LNN group, underlying the immune gene expression changes observed at the transcriptome level (Fig. 6- B, C).

### 3.5 Impairments in the mitochondrial function, autophagy and apoptotic signalling in 3-month-old male offspring on gestational protein restriction

The potential impact of immune-related gene upregulation observed at 21 days in male offspring mice on gestational protein restriction was assessed on mitochondrial function, oxidative stress levels, and inflammation at 3 months of age.

**Fig. 7:**
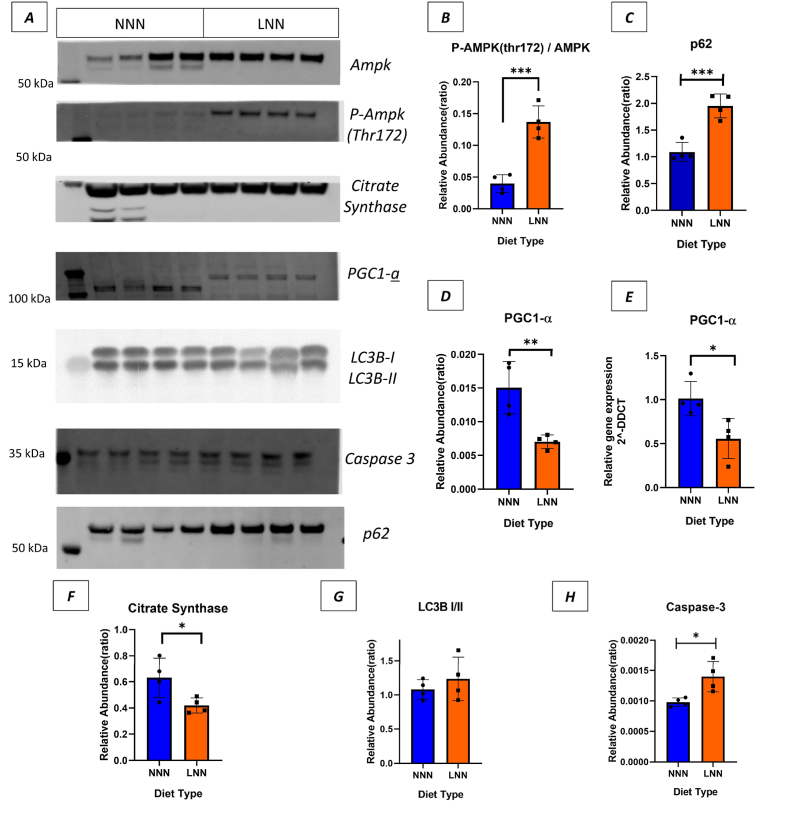
**Gestational protein restriction influences energy metabolism and mitochondrial function markers in 3-month-old offspring male mice.** **(A***) Western blot results for AMP-activated protein kinase (AMPK), p-AMPK (Thr172), Citrate synthase, (Pparg coactivator 1 alpha) Pgc1- α, LC3B–I and LC3B-II, Caspase-3, and p62 respectively. Quantification and statistical analysis of protein relative abundance ratio in graphs Thr172/Ampk****(B)****, p62****(C)****, Pgc1- α****(D),****Citrate Synthase****(F)****LC3B–I and LC3B-II****(G),****Caspase-3****(H).****Rt-qPCR analysis of Pgc1- α****(E).****GAPDH for Citrate Synthase, Pgc1- α* (Supplementary Fig. 1–C), *Ponceau staining were used for caspase-3 and P62 as a protein loading control* ((Supplementary Fig. 1–C). *∗ indicates p < 0.05, ∗∗ indicates p < 0.01, ∗∗∗ indicates P < 0.001. Student’s t-test used as a statistical test****(n = 4).***

We measured PGC1-α relative protein abundance (p < 0.01) and gene expression (p < 0.05), which were both significantly lower, indicating impaired mitochondrial biogenesis (Fig. 7- A, D, E). It is worth noting that a shift in the molecular weight of PGC-1-α was observed in the LNN group. This is possibly due to posttranslational modifications that could be occurring in the LNN group. Citrate synthase relative abundance, a marker of mitochondrial content, was also reduced, further supporting evidence of mitochondrial dysfunction (Fig. 7- A, F). Consistent with these findings, in the LNN group, there was a significant increase (p < 0.001) in the phosphorylated AMPK (Thr172) to total AMPK ratio (Fig. 7- A, B), indicating activation of pathways related to cellular metabolic energy stress.

Next, autophagy markers were analysed, showing a slight but non-significant increase in the LC3B–I/II ratio (*p* > 0.05) (Fig. 7-A, G). However, p62 relative abundance, a marker of autophagic activity, was also increased, supporting evidence of disrupted autophagic processes. (Fig. 7-A, C). Furthermore, Caspase-3 protein expression was significantly elevated (p < 0.05), indicating enhanced apoptotic signalling (Fig. 7-A, H).

## 4 Discussion

Maternal protein deficiency (MPD) continues to be a global health concern [9,10] hence investigating the underlying mechanisms impacting offspring health and well-being is of prime importance. In this study, we investigated the effects of a gestational low-protein diet on soleus and gastrocnemius muscles in male offspring to establish its impact on skeletal muscle development and function in weaned and adult male offspring. A MPD-induced SGA phenotype in offspring has been linked previously to raised oxidative stress and disruptions in metabolic regulation, as demonstrated in multiple animal models [32]. Our study demonstrates that although the protein restricted group was nursed by normally fed dams, they exhibited reduced body and SOL muscle weights at the end of the lactation period. Following post-weaning diet with a standard 20 % protein intake, these animals experienced a catch-up growth, resulting in similar body and muscle weights to control group by 3 months of age. It is well established that catch-up growth is associated with reduced lifespan [33] and metabolic dysfunction in mice, rats and humans [18,34].

We thus examined SOL muscle histologically to determine changes in fibre number, CSA, and fibre type remodelling in both 21 day-old and 3 months-old male mice. Confortim et al. (2015) demonstrated that male offspring exposed to an isocaloric maternal low-protein diet during both gestation and lactation displayed smaller CSA of soleus muscle fibres at 12 months of age, despite no significant change in total fibre number in rats [35]. Interestingly, the same group in another study also reported that by day 21 postnatally, the protein restricted group showed significantly increased fibre number in the rat soleus muscle, even though muscle weight and CSA was reduced [36]. In contrast to these previous findings in rats, our results revealed that LNN mice have decreased fibre number at 3 months of age. Our group previously published that a gestational low-protein diet had no significant effects on extensor digitorum longus (EDL) muscle fibre diameter in 21 day-old mixed-gender offspring mice [37]. On the other hand, our group demonstrated that lifelong protein restriction leads to a reduction in type-IIa fibre number in the soleus muscle of 18-month-old mice [15] and a decrease in CSA in the tibialis anterior muscle at 3 months of age [16]. Consistently, the present study showed that gestational protein restriction led to a reduction in type-IIa fibre number at 21 days of age, which was restored by 3 months old. Nevertheless, no significant differences in CSA were detected at either time point. These findings may reflect an adaptive remodelling process in muscle fibre phenotype, as nutritional changes may promote alterations in muscle fibre type specific composition [38].

To understand the underlying changes in skeletal muscles of LN mice, RNA-Seq profiling was performed; this data showed that adaptive immunity-related genes were upregulated in the LN group suggesting active immune response in protein restricted mice (21 days). Furthermore, Gene Ontology Biological Process (GO-BP) analysis revealed a significant enrichment in pathways including T cell activation, and the adaptive immune response. On the other hand, a study demonstrated that an isocaloric low-protein diet during gestation in mice resulted in altered mitochondrial gene expression in the new-born offspring [20]. These changes could be explained by increased protein intake during lactation following cross-fostering after birth [39,40], as studies have shown that maternal nutrition can alter the composition of breast milk both in humans and rodents [41,42].

Macrophages are crucial part of the immune system and T cells plays pivotal role in recruiting and activating them [43,44]. Therefore, we next analysed the macrophage infiltration as a potential downstream effect of adaptive immune changes in SOL and GAS muscles of 3 months old male offspring. Interestingly, we observed an increased trend for infiltration of CD68^+^ macrophages in the SOL muscle. Mouse macrophages are relatively small cells, typically ranging from 10 to 20 μm in diameter [45]; hence their detection in 8–10 μm-thick muscle sections may be limited. To address this limitation, we also assessed CD68 protein abundance in the GAS muscle, which revealed an increased level. These findings suggest a sustained immune response accompanied by ongoing inflammation. NF-κB plays a central role in regulating immune function and inflammation [46]. RelA (p65), a key subunit of the NF-κB complex, is essential for controlling inflammatory responses, cellular proliferation, and programmed cell death [47]. A previous study showed that gestational low-protein diet induces oxidative stress, compensatory antioxidant responses, and inflammation, potentially regulated by NF-κB signalling in 12 months old rats [17]. With this in mind, we investigated p65 protein abundance in GAS muscle, which was increased in LNN group. This finding supports that the muscles of LNN mice experience ongoing inflammation [48]. In addition, NF-κB regulates genes that control cellular ROS levels, while ROS can, in turn, have either inhibitory or stimulatory effects on NF-κB signalling pathways. This bidirectional interaction is linked not only to inflammation and immune responses but also to antioxidant regulation [49,50]. Indeed, we found that the antioxidant balance in GAS muscle was disrupted, as indicated by decreased SOD2 and increased HO-1 levels, alongside increased protein carbonyl level, which are irreversible products of ROS and a hallmark of oxidative damage [51,52].

The potential link between immune activation in early postnatal life and later oxidative stress and inflammation was investigated by assessing energy metabolism markers in GAS muscles at 3 months of age. Our findings indicated a decrease in both PGC1-α and citrate synthase levels, highlighting impaired mitochondrial biogenesis [26] and reduced mitochondrial content [53], respectively. This was accompanied by a significant increase in the phosphorylated AMPK to total AMPK ratio, suggesting activation of energy stress signalling [54]. A recent study also supports that maternal low protein diet causes impaired energy metabolism in 4 month-old rat offspring [55]. These results reflect a disrupted mitochondrial regulatory network in response to early-life nutritional challenges. Mitochondrial dysfunction, induced by inflammation and oxidative stress, is a crucial cause of skeletal muscle atrophy [56]. We previously demonstrated that lifelong protein restriction induces skeletal muscle atrophy [57]. Moreover, we also analysed autophagy markers in the LNN mice, which showed increased levels of p62 and caspase-3, while the LC3B–I/II ratio remained unchanged in GAS muscle. These findings indicate a potential impairment in autophagic flux alongside activation of apoptotic signalling pathways.

This study has some limitations. These include a relatively small sample size and the inability to assess macrophage polarisation through histological methods. The individual food intake could not be measured, as the animals were housed in groups. In this study we focused on the soleus and gastrocnemius muscles; inclusion of other fast-twitch muscles such as the tibialis anterior and extensor digitorum longus (EDL) would have allowed for a more comprehensive comparison.

**Fig. 8:**
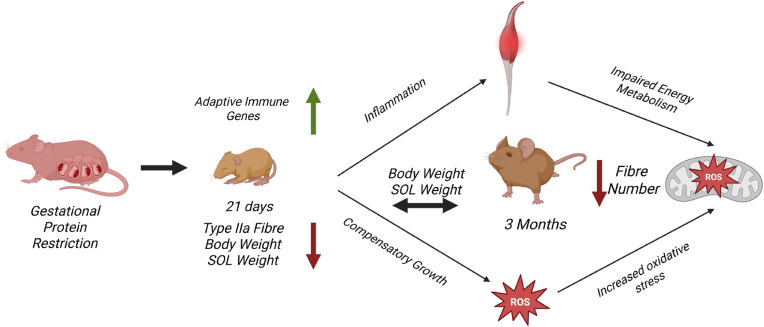
**Graphical Abstract Effect of gestational protein restriction followed by a normal protein diet on skeletal muscle in male offspring.** (*Created in BioRender. ALTINPINAR, A (2025)*https://BioRender.com/Xvfugyh*).*

In conclusion, our study demonstrates that a gestational low-protein diet led to a reduced body weight, soleus muscle mass, and altered type-IIa fibre proportion, whilst upregulating adaptive immune-related genes at weaning. These changes promoted increased inflammation and were followed by catch-up growth in young mice fed a normal protein diet. Additionally, mitochondrial dysfunction and impaired autophagy were evident. Even though fibre remodelling appeared to recover following the normal diet, the total fibre number reduced in SOL muscle at 3 months of age. Our findings thus reveal novel immune-related mechanisms of how a gestational low-protein diet affects male offspring skeletal muscles at early ages. Future studies are warranted to address the long-term consequences of this low protein diet induced molecular remodelling (see Fig. 8).

## CRediT authorship contribution statement

**Atilla Emre Altinpinar:** Writing – original draft, Software, Methodology, Investigation, Formal analysis, Data curation. **Moussira Alameddine:** Methodology, Data curation. **Ufuk Ersoy:** Methodology, Data curation. **Ioannis Kanakis:** Writing – review & editing, Supervision, Methodology, Funding acquisition. **Vanja Pekovic-Vaughan:** Writing – review & editing, Supervision. **Susan E. Ozanne:** Writing – review & editing, Methodology. **Katarzyna Goljanek-Whysall:** Writing – review & editing, Supervision, Funding acquisition. **Aphrodite Vasilaki:** Writing – review & editing, Supervision, Project administration, Methodology, Funding acquisition, Data curation.

## Funding

A.V., I.K. and K.G-W. disclose support for the research of this work from 10.13039/501100000268Biotechnology and Biological Sciences Research Council (grant numbers BBSRC; (BB/P008429/1 and BB/W018276/1). A.E.A. and U.E disclose support for the research of this work from the Republic of Türkiye Ministry of National Education. The authors disclose support for this work from the University of Liverpool.

## Declaration of competing interest

None.

## Data Availability

The raw RNA-Seq data have been deposited in NCBI Gene Expression Omnibus (GEO) database under accession number GSE307422.
