# Supplementary material for: Gestational low-protein diet impairs mitochondrial function and skeletal muscle development by inducing immune responses in male offspring
Source: Redox Biol. 2025 Oct 10;87:103890. doi: 10.1016/j.redox.2025.103890 (PMC12550721; doi:10.1016/j.redox.2025.103890)
Supplement: Multimedia component 1 [file mmc1.pptx]

## Slide 1
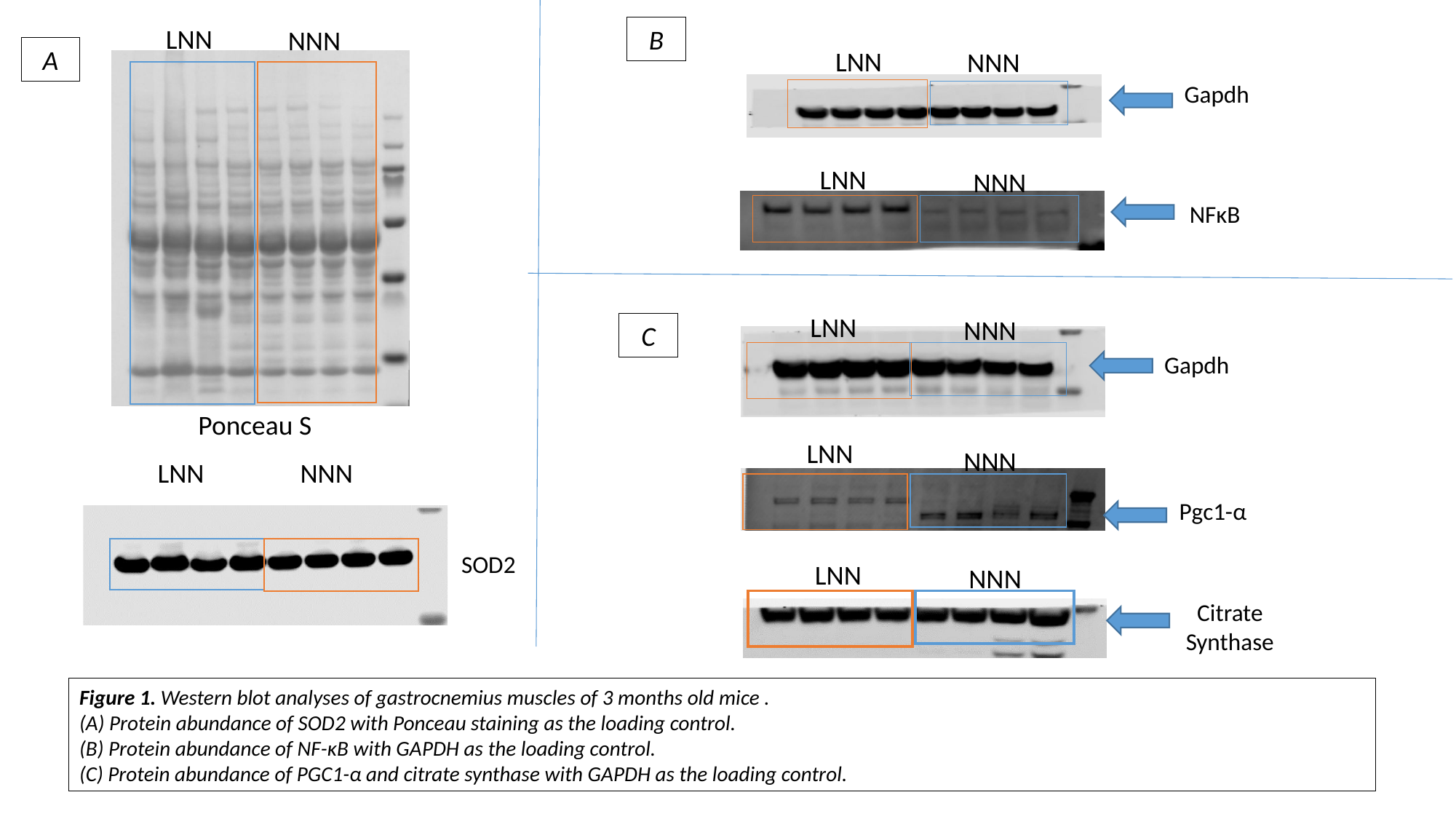

LNN
B
NNN
A
LNN
NNN
Gapdh
LNN
NNN
NFκB
LNN
NNN
C
Gapdh
Ponceau S
LNN
NNN
LNN
NNN
Pgc1-α
SOD2
LNN
NNN
Citrate Synthase
Figure 1. Western blot analyses of gastrocnemius muscles of 3 months old mice .
(A) Protein abundance of SOD2 with Ponceau staining as the loading control.
(B) Protein abundance of NF-κB with GAPDH as the loading control.
(C) Protein abundance of PGC1-α and citrate synthase with GAPDH as the loading control.

## Slide 2
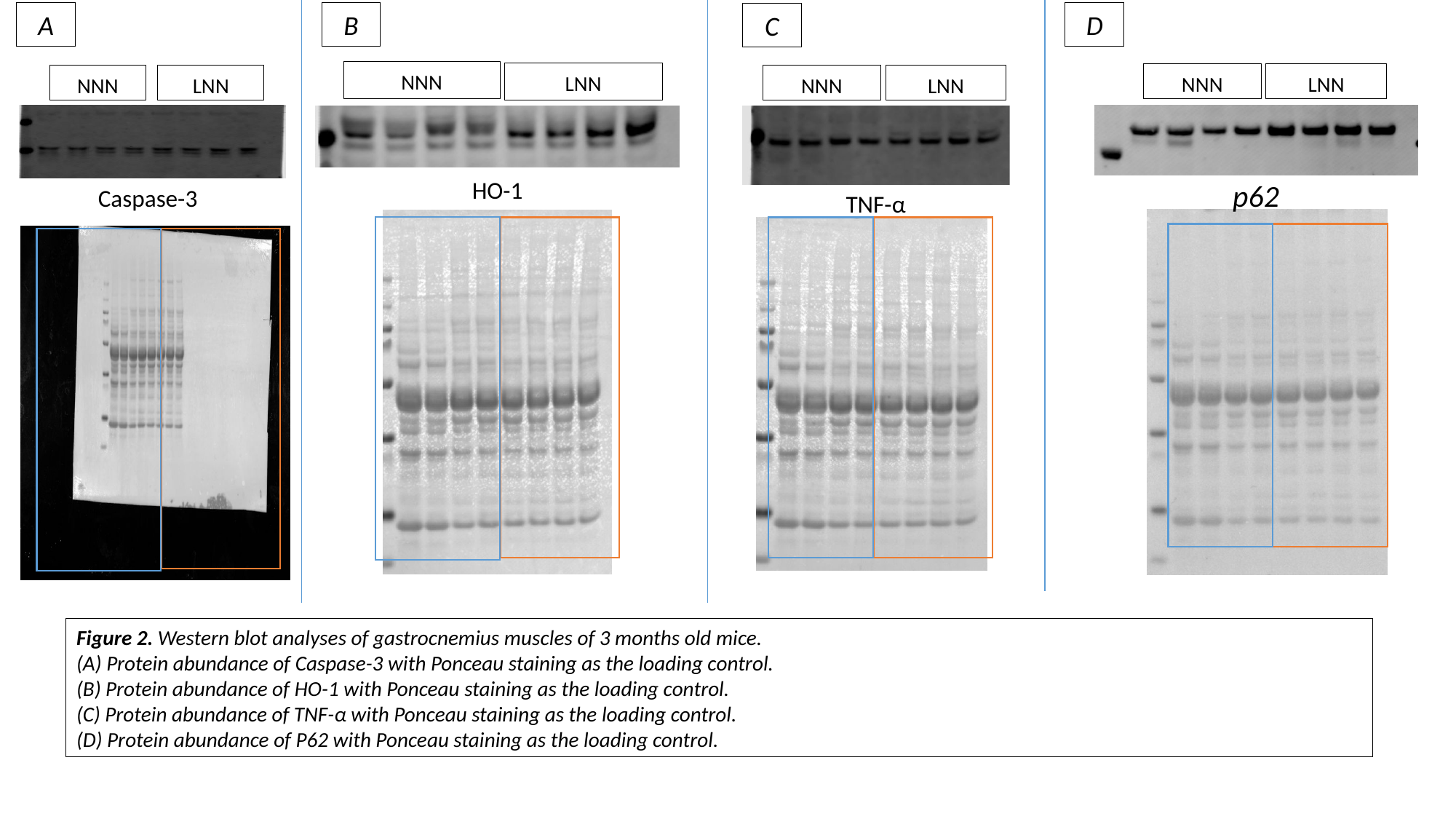

A
B
D
C
NNN
LNN
NNN
LNN
NNN
NNN
LNN
LNN
HO-1
p62
Caspase-3
TNF-α
Figure 2. Western blot analyses of gastrocnemius muscles of 3 months old mice.
(A) Protein abundance of Caspase-3 with Ponceau staining as the loading control.
(B) Protein abundance of HO-1 with Ponceau staining as the loading control.
(C) Protein abundance of TNF-α with Ponceau staining as the loading control.
(D) Protein abundance of P62 with Ponceau staining as the loading control.

## Slide 3
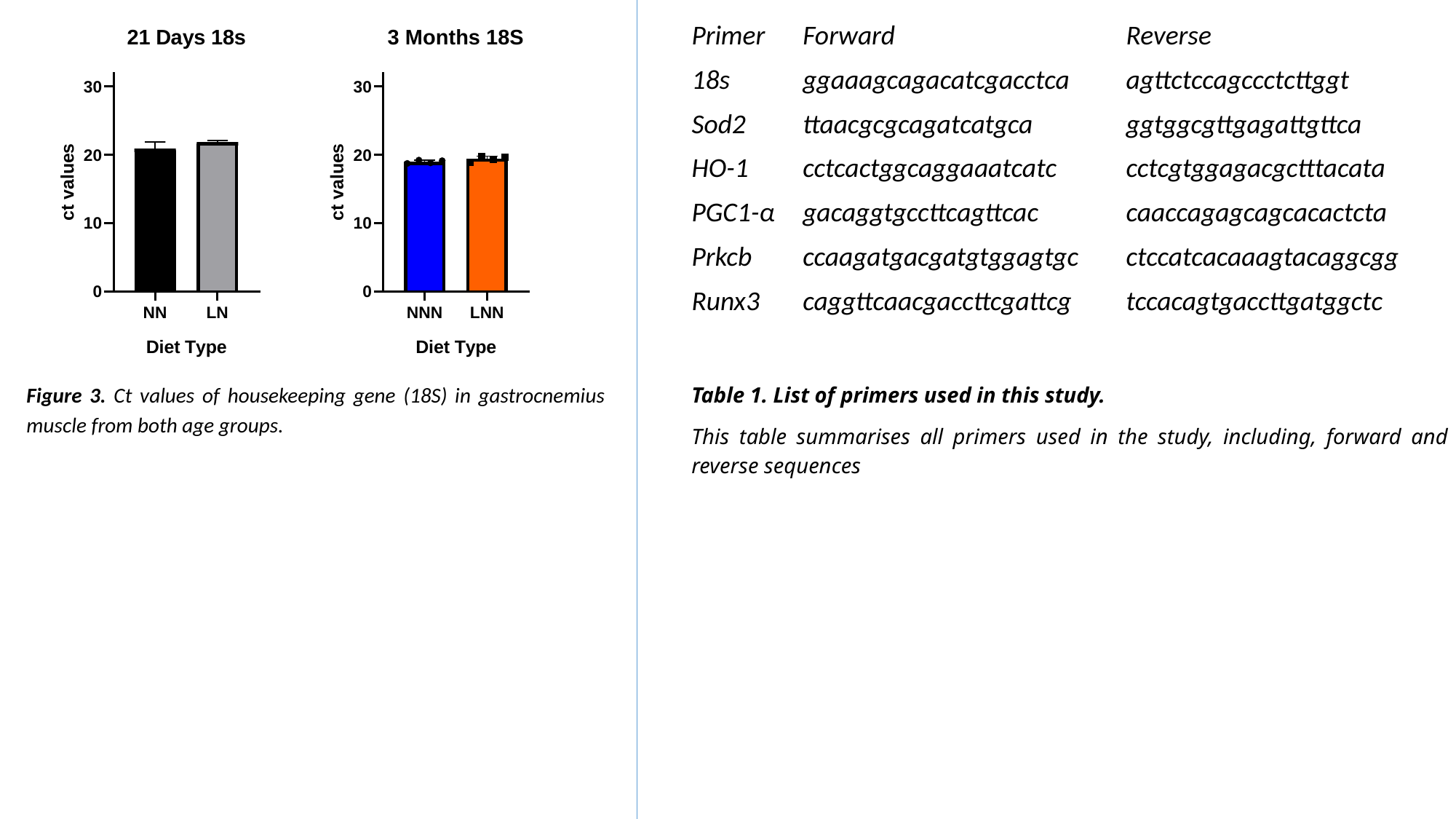

| Primer | Forward | Reverse |
| --- | --- | --- |
| 18s | ggaaagcagacatcgacctca | agttctccagccctcttggt |
| Sod2 | ttaacgcgcagatcatgca | ggtggcgttgagattgttca |
| HO-1 | cctcactggcaggaaatcatc | cctcgtggagacgctttacata |
| PGC1-α | gacaggtgccttcagttcac | caaccagagcagcacactcta |
| Prkcb | ccaagatgacgatgtggagtgc | ctccatcacaaagtacaggcgg |
| Runx3 | caggttcaacgaccttcgattcg | tccacagtgaccttgatggctc |
Figure 3. Ct values of housekeeping gene (18S) in gastrocnemius muscle from both age groups.
Table 1. List of primers used in this study.
This table summarises all primers used in the study, including, forward and reverse sequences
